# Use of a sperm FcR assay in intracytoplasmic sperm injection improves day 5 good quality blastocyst yield

**DOI:** 10.1007/s10815-026-03978-z

**Published:** 2026-07-25

**Authors:** Rebecca Holmes, Nadir Ciray, Kristy Savage, Hossein Yazdekhasti, Zimu Chen, John Walsh, Barb Cohen, Larry Lipshultz, Jason E. Swain

**Affiliations:** 1 CCRM Boston, Chestnut Hill, USA; 2 CCRM Fertility Network, Lone Tree, USA; 3 OvoScore, Rockville, USA; 4 Arex Life Sciences, Watertown, USA; 5 Department of Urology, Baylor College of Medicine, Houston, USA

**Keywords:** Sperm, Function, Fc receptor, Capacitation, ICSI

## Abstract

**Purpose:**

The Kinetix FcR binding assay results correlate to male fertility in intrauterine insemination. Kinetix measures temporal expression of a sperm Fc receptor as sperm capacitate and become fertile, which in healthy ejaculates occurs over time in sequential groups. We evaluated the ability of Kinetix to predict ICSI outcomes.

**Methods:**

A prospective observational study was conducted on human semen samples during ICSI procedures to measure the changing proportion of sperm expressing the FcR over time. Semen samples were then categorized as “Fertile,” “Subfertile,” or “Infertile” depending on the pattern of functional sperm populations. The fertilization rate, yield of day 5 total, and day 5 good quality blastocysts (GQB) were quantitated.

**Results:**

We found 2.9 times more day 5 GQB/2PN (2 pronuclei (2PN) visible) using sperm identified as Fertile compared to the Infertile group. No day 5 GQB were produced in 65% of cycles using sperm from the Infertile group. Day 5 GQB were produced in 65% of cycles using sperm from the Subfertile and 77% from the Fertile group of ejaculates. Male and female demographics, rapidly progressive motility, and sperm concentration were not statistically different across groups.

**Conclusions:**

Notably, the sperm functional characteristics revealed by the Kinetix Sperm FcR Assay were irrespective of semen quality as defined by current semen analysis techniques. It is likely that a higher Kinetix score reflects the presence and ultimate injection of higher quality sperm, which improves the yield of day 5 good quality blastocysts.

**Supplementary information:**

The online version contains supplementary material available at https://doi.org/10.1007/s10815-026-03978-z.

## Introduction

With the advent of intracytoplasmic sperm injection (ICSI), a powerful tool was made available for the treatment of male factor infertility during assisted reproduction. However, several reports suggest that ICSI may now be overused, and its application may unnecessarily promote more expensive treatments and result in a deprioritization of research focused on further elucidation of important biological mechanisms of sperm function [1–3].

Historically, sperm assays were utilized in an attempt to elucidate sperm fertilization capacity or other functional properties. Many of these tests have largely been abandoned or rendered uninformative. This development is due, in part, to the advent of direct injection of sperm into the oocyte via ICSI. However, there remains a high level of unexplained infertility regardless of the procedure used, creating the need for additional tools to better assess, monitor, diagnose, and treat sperm-based infertility. Methods to further assess sperm function and quality could still be beneficial, even with ICSI.

For normal fertilization to occur, sperm must undergo a capacitation process. Originally discovered by Austin [4] and Chang [5], capacitation is not completely understood. Hyperactivated motility and acrosomal changes culminating in the ability to undergo the acrosome reaction are a hallmark of capacitation, but this is not an exhaustive accounting of essential processes. Other cellular and molecular changes accompany sperm maturation and the ability to successfully fertilize a mature oocyte [6]. Most relevant here are studies by the Eisenbach laboratory [7], which demonstrated that human sperm activate in sequential waves which initiate at varied times post-ejaculation, and the activated groups are short-lived (~ 1–2 h). A consequence of this process is a fluctuating pattern of higher and lower fertility within an ejaculate, as active sperm groups gain and lose fertility over time before finally senescing altogether.

These fertility fluctuations can be detected by an assay that monitors changes in sperm populations exhibiting specific protein levels over time. When a cycle of capacitation occurs, the Gerton laboratory showed that different proteins become detectable on the sperm acrosomal cell surface, from which many are then shed [8]. A protein both detectable on the acrosome [9] and in seminal plasma [10] is an Fc receptor (FcR). Its expression was correlated with fertility when Sedor et al. [11] reported men with lower levels of functional FcR in semen are less fertile. An explanation for this was proposed by Sakin-Kaindl et al. [12], who suggested FcR enables sperm to resist female immune attack by suppressing antibody-dependent cellular cytotoxicity. On the genomic level, a transcript for an FcR was found at 20-fold higher levels in sperm producing IUI pregnancy compared to those that failed [13].

Subsequent development and preliminary testing of a sperm surface proteomic assay for functional FcR, run multiple times on an ejaculate at 30-min intervals, identified several distinct temporal patterns of sperm populations exhibiting specific protein levels [14–16]. These distinct patterns were hypothesized to reflect the sequence of sperm populations exhibiting specific protein levels that occur as groups of short-lived capacitating sperm arise, mature, and senesce, as described by Cohen-Dayag et al. [7].

## Materials and methods

### Study design

A prospective observational cohort study was conducted. Fresh semen samples of the procedure ejaculate were obtained from the materials for discard from patients undergoing ICSI treatment. Use of discarded materials was via approval from the Western Institutional Review Board-Copernicus Group (no. 20142468).﻿ After routine preparation of semen samples, aliquots of the remaining samples were analyzed with the Kinetix Sperm Fc Receptor (FcR) Binding Assay (Arex Life Sciences, Watertown, MA). The Kinetix assay detects changes in expression of an acrosomally-expressed functional FcR as a marker of capacitation and identifies distinct temporal patterns that are associated with the functional quality of sperm capacitation in a semen sample [14, 16].

### ICSI procedure

**Fig. 1:**
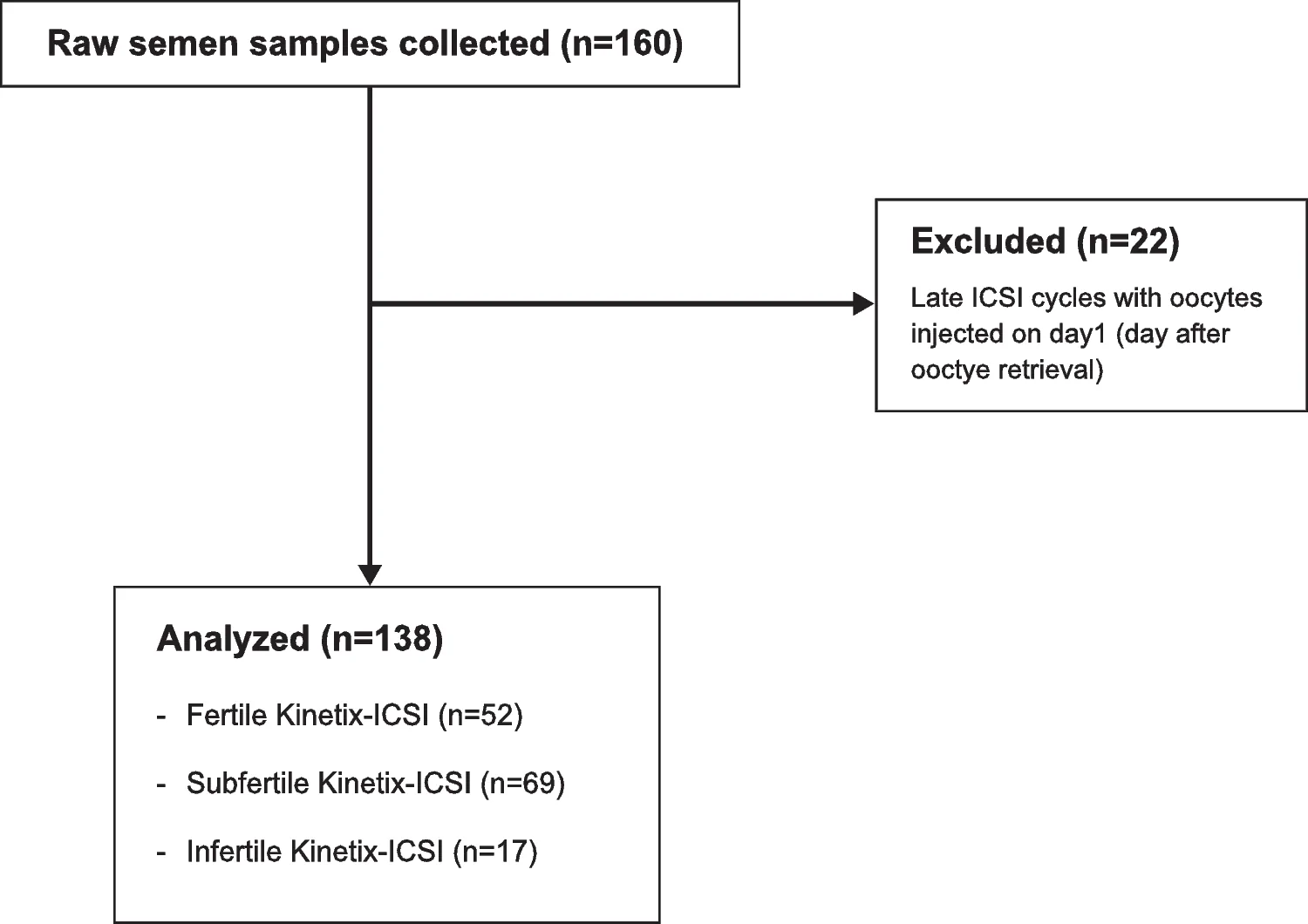
ICSI analysis strategy and distribution of Kinetix scores

From a total of 160 ICSI cycles, only cycles with ICSI occurring on day 0, the day of oocyte retrieval, were included, resulting in assessment of 138 cycles (Fig. 1). Female patient infertility included recurrent implantation failure, recurrent pregnancy loss, diminished ovarian reserve, and ovulatory dysfunction. Male demographics included age, sample volume, sperm concentration, and rapidly progressive motility (assessed according to the World Health Organization, 6th edition (+ 4 and + 3, WHO 6th edition: rapidly progressive; ≥ 25 µm/s: spermatozoa moving actively, either linearly or in a large circle, covering a distance from the starting point to the end point, of at least 25 µm, or ½ tail length, in 1 s). Morphology was not assessed. Female demographics included female age, anti-Mullerian hormone (AMH), and day 3 follicle stimulating hormone (FSH) levels. Patients with poor oocyte quality were excluded from the study. Poor oocyte quality was defined by the presence of any of the following: vacuoles, smooth endoplasmic reticulum aggregates, fragmented polar bodies, or overly compliant oocyte membrane elasticity. Eggs with hard or thick zonas were used in the normal fashion, and zona status was recorded as an observation. Extended embryo culture was conducted in drops of Sage BM (Origio, Cooper Surgical, Trumbull, CT) overlayed with either Ovoil Heavy (Vitrolife, Sweden) or Hypure® Oil Heavy (Kitazato, Japan) for up to 7 days at 37 °C in 5.0% O_2_ and 6.5% CO_2_.

ICSI was performed with standard protocols without alteration of the procedure. Outcome data described below were recorded to correlate to the Kinetix assay’s sperm [16] functionality results from the same sperm sample. The fertilization rate and production of day 5 blastocysts per normally fertilized oocyte, defined as formation of 2 pronuclei (2PN), were recorded. Standard Gardner Grading Scale criteria (≥ 3BB [17]) were used to score resulting blastocysts across days 5–7. Outcomes were compared to sperm expression patterns (Fertile, Subfertile, and Infertile) to determine if associations existed. Standard semen assessments were performed per routine ICSI Standard Operating Procedure (SOP) for volume, rapidly progressive motility, and concentration on all samples. Morphology was not assessed. Fresh semen samples of the procedure ejaculate were obtained from the materials for discard from patients undergoing ICSI treatment. Use of discarded materials was via approval from the Western Institutional Review Board-Copernicus Group (no. 20142468).

Statistical analysis was performed by analysis of variance (ANOVA) followed by Tukey’s honestly significant difference (HSD) post-hoc test for significant differences among groups after checking for distribution of the numerical data for normality and by chi-square test for categorical data. A *p* value of < 0.05 was considered significant.

### Kinetix sperm FCR assay

This sperm function assay measures temporal changes in expression of a functional FcR, one capable of binding the Fc region of antibodies, as sperm capacitate and gain fertility. A total of 35 uL of semen were analyzed in sequential assays performed at 30-min intervals with successive 5 uL aliquots of the laboratory semen sample over a 3-h period. Temporal expression patterns of FcR were analyzed by flow cytometry (BD Accuri c6 Flow Cytometer). The resulting temporal expression patterns from each ejaculate were analyzed with a proprietary algorithm that interprets multiple features of the signal to generate a score that represents the capacitation status of sperm in an ejaculate. This enables the Kinetix assay [16] to produce a numerical quality score for each ejaculate’s capacitation ability with ejaculates’ sperm function categorized as Fertile, Subfertile, or Infertile sperm. For each patented Kinetix FcR assay, a microfuge tube was prepared with a cocktail consisting of the following agents: 100 µL of commercial antibody diluent containing bovine serum albumin (ThermoFisher Scientific, Waltham, MA); 20 µL of a primary antibody solution made by dissolving lyophilized rabbit serum (BD Biosciences, Franklin Lakes, NJ), reconstituted at the manufacturer’s recommended dilution into phosphate buffer (MP Biomedicals LLP, Solon, OH); and 1 µL of a 1:10 (v/v) dilution of a solution of DRAQ 5 (ThermoFisher Scientific, Waltham, MA). To this, 5 µL of the laboratory discard semen specimen was added plus 5 µL of a secondary antibody solution, a polyclonal Alexa Fluor 488 conjugated goat anti-rabbit antibody (ThermoFisher Scientific, Waltham, MA), used at the concentration supplied by the manufacturer. After a 20-min incubation at ambient temperature, 1 mL of phosphate buffer (MP Biomedicals LLP, Solon, OH) was added, and the tube was then mixed by inversion and centrifuged at 2000 × g for 30 s. The supernatant was then aspirated, and 300 µL of phosphate buffer was added to resuspend the sperm pellet. The sample was then analyzed on an Accuri C6 cytometer (Accuri, Lansing, MI; now BD, Franklin Lakes, NJ). The temporal FcR expression pattern for each ejaculate was evaluated by graphing changes in FcR expression with seven sequential time points of 5 µL subaliquots from the specimen at 30-min intervals. The percentage of sperm cells expressing FcR, as detected by flow cytometry, was recorded at each time point to produce the temporal FcR expression pattern for the ejaculate. This pattern was superimposed on the temporal timing of egg injection to determine whether the sperm population’s FcR expression was associated with the yield of day 5 GQB. FcR expression patterns were analyzed by a proprietary algorithm.

## Results

### Patient characteristics

**Fig. 2:**
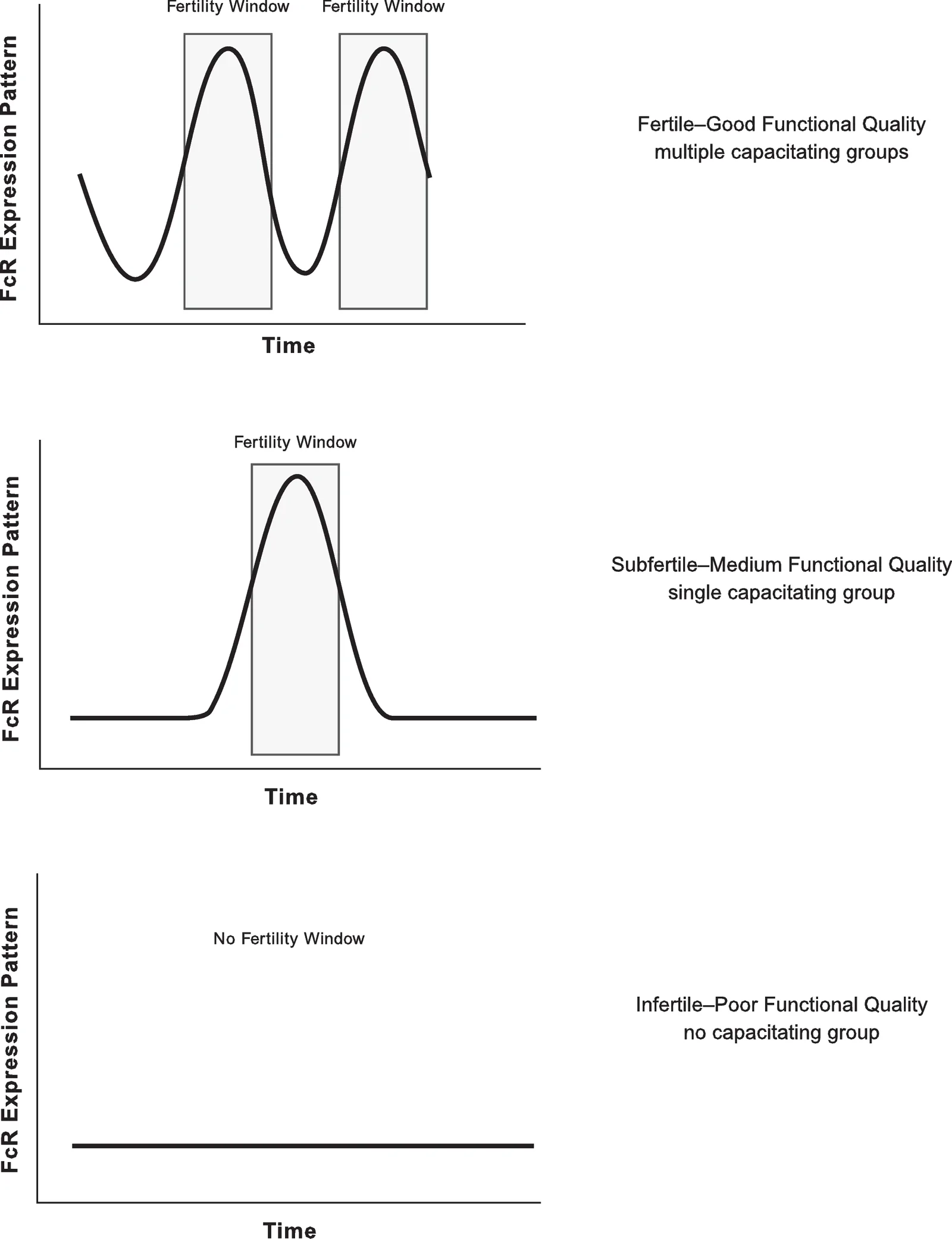
Kinetix assay score groups observed by assay, reflecting the timing and quality of capacitating sperm groups in the tested ejaculate

As an initial point of reference, the cohort of *n* = 138 ejaculates used in this evaluation were assessed against standard semen analysis parameters (Supplementary Table 1). They were analyzed as outcomes for each patient whose eggs were injected with sperm from ejaculates having sperm function categorized by functional scores of Fertile, Subfertile, or Infertile (Fig. 2). The majority of the samples had normal semen parameters when evaluated against the World Health Organization (WHO) 6th edition reference ranges including rapidly progressive motility (+ 3 and + 4 motility) as shown in Supplementary Table 1. Several female side parameters were analyzed as well including female age, Anti-Mullerian hormone (AMH) and day 3 follicle stimulating hormone (FSH) as shown in Supplementary Table 2. There were no statistical differences between Kinetix score groups with respect to female age and standard semen parameters as well as rapidly progressive motility based on one-way ANOVA statistical calculations. Similarly, there were no statistical differences in the yield of day 6 or day 7 good quality blastocysts (Supplementary Table 3).

### Kinetix assay results

The Kinetix assay demonstrated three distinct functional score groups regarding temporal FcR expression (Fig. 2), which were defined as “Fertile,” “Subfertile,” and “Infertile.” These scores reflect the functional effectiveness of sperm to capacitate in repeated groups over the timeframe of repeat measurements.

Stratification of ejaculate functional quality by score group enabled evaluation of each group’s association with semen analysis parameters, fertilization rates, yields of day 5 good quality blastocysts, and failure to produce day 5 good quality blastocysts. The association of assay grouping with outcome was independent of assessed male and female parameters (Supplementary Tables 1 and 2). This is consistent with the assay correlation to outcome measures being attributed to Kinetix scores.

Next, oocyte fertilization rates and blastocyst yield (total and day 5 good quality) per normally fertilized oocyte were evaluated. There was no statistically significant correlation between Kinetix score group and fertilization rate, defined as production of two pronuclei. Each assay group was then evaluated for yield of total and good quality blastocysts. Yield of day 5 GQB was associated with the Kinetix Score (Table 1). A statistically significantly higher yield of day 5 GQB per 10 eggs fertilized normally (2 pronuclei (2PN) visible) was obtained, with an average of day 5 GQB/10 eggs (2PN) of 2.9 in the Kinetix Fertile score group, 2.0 in the Subfertile group, and 1.0 in the Infertile group.

**Table 1:** Day 5 good quality blastocyst yield per 10 normally fertilized oocytes in each Kinetix assay score group (one way ANOVA *p* = 0.008, followed by Tukey HSD post-hoc test where *p* = 0.01* when Kinetix score groups Fertile and Infertile are compared)

Kinetix score	Cycles (*n*)	#Oocytes retrieved, average	#Oocytes injected, average	Mean day 5 GQB/10 eggs Fert. norm
Fertile	52	12.4 ± 6.5	10.7 ± 5.7	2.9 ± 2.4*
Subfertile	69	13.7 ± 7.3	10.5 ± 5.9	2.0 ± 2.2
Infertile	17	11.0 ± 7.2	9.7 ± 6.0	1.0 ± 1.6*

*D5 GQB*, day 5 good quality blastocysts

High sperm function in an ejaculate was not only associated with significantly higher yields of day 5 good quality blastocysts but was also statistically significantly associated with a lower percentage of cycles lacking formation of day 5 GQB (Table 2). No day 5 GQB were produced by sperm in the Infertile group 65% of the time. This failure rate was lower at 23% in the Fertile group. The lower yield of day 5 GQB as Kinetix score declined, reflecting poorer functional quality, was associated with a higher failure rate of sperm exhibiting poor functional quality to produce day 5 GQB.

**Table 2:** Number of cycles producing no day 5 good quality blastocysts (D5 GQB) per normally fertilized oocyte in each Kinetix assay score group (*p* = 0.007, chi-square test among three groups)

Kinetix score	Number of cycles with no D5 GQB/total	% cycles with no D5 GQB	*p* value
Fertile	12/52	23	
Subfertile	24/69	35	
Infertile	11/17	65	

The Infertile group had significantly more cycles without day 5 GQB than either the Fertile* or Subfertile** groups (*p = 0.003 for Fertile vs Infertile and **p = 0.03 for Subfertile vs Infertile, chi-square test)

## Discussion and conclusion

Production of good quality blastocysts is critical to ART success. However, its prediction from the sperm quality measures has been difficult. While various studies have examined the impact of sperm DNA fragmentation on ART outcomes and embryo development [18, 19], these data are far from conclusive. Additionally, these assays are not based on the sample used for the actual procedure, so predictive value on clinical outcomes can be difficult.

This study demonstrates that applying the Kinetix assay in ICSI, through detection of temporal FcR expression patterns on sperm, can identify ejaculates associated with significantly higher day 5 good quality blastocyst yields per fertilized oocyte. Sperm with function scored as Subfertile or Fertile have increasing yields of day 5 GQB, respectively. Sperm with Infertile function by this measure have significantly reduced ability to generate good quality day 5 blastocysts, despite good fertilization rates. In a 2022 report on ART cycles, male factor only was reported in 16.5% of cases [20]. The Kinetix assay quality score classified 12.3% of samples as Infertile quality ejaculates. These were associated with no day 5 GQB in 65% of the cycles. The sperm functionality distinguished by the Kinetix assay was not detectable with standard semen analysis. Of note, sperm morphology was not assessed or compared in this study.

**Fig. 3:**
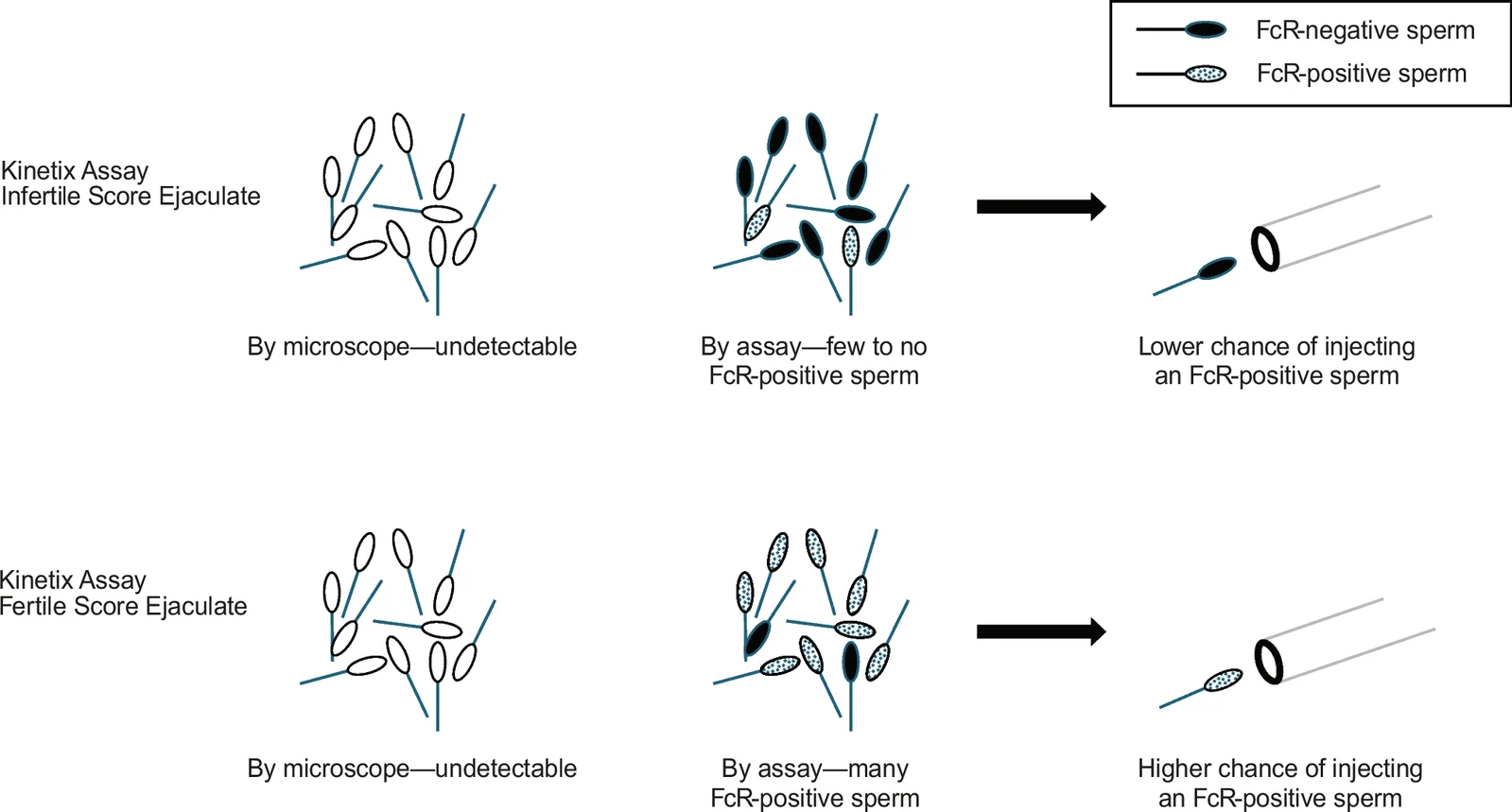
The probability of injection of a FcR-positive sperm increases as Kinetix score improves

The results of the present study are in accordance with earlier findings [16, 21–23]. Capacitation was suggested as a marker for improved blastocyst formation in a human sibling oocyte study where injections with a higher proportion of capacitated sperm produced a significantly higher yield of day 5 good quality blastocysts than injections with a lower proportion of capacitated sperm in the injection pool [21]. FcR expression in the Kinetix assay is followed by FcR shedding [16, 22], a marker of acrosomal exocytosis and therefore of capacitation [23]. In addition, use of the Kinetix assay in a preliminary study in humans reported an increase in pregnancy rates from 21 to 42% in a group of patients where some couples with female factor issues were excluded, raising the baseline cycle success rate [16]. Results of the present study corroborate these findings by demonstrating that selection and utilization of human sperm from ejaculates with Fertile function or even Subfertile function—having a higher proportion of FcR-positive sperm in the injection pool—enable improvement in day 5 good quality blastocyst yield in ICSI cycles as shown in the working model (Fig. 3).

**Fig. 4:**
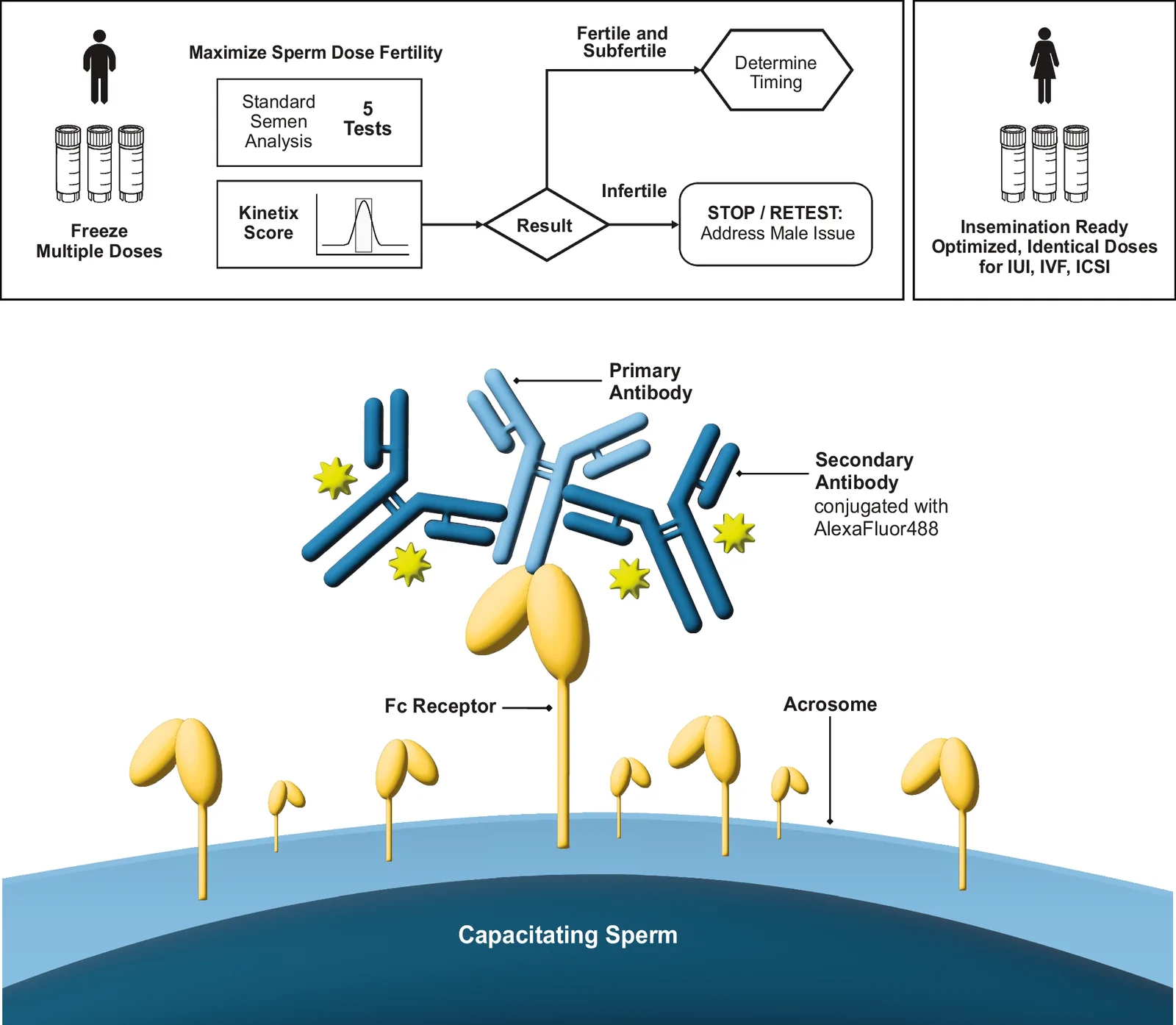
Analysis of cryopreserved ejaculates to assess quality and choose the best sperm prior to female treatment and mechanism of assay detection on capacitating sperm of functional Fc Receptor, i.e., a ligand capable of binding the Fc region of antibodies (reproduced from Cohen B et al. (2025) Systems Biology In Reproductive Medicine 71:1, 630–645, https://doi.org/10.1080/19396368.2025.2590747 under a CC BY-NC-ND 4.0 license)

The clinical benefit for semen analysis with the Kinetix assay is the potential to improve the clinical outcomes of ICSI by significantly improving the yield of day 5 good quality blastocysts with a yield of almost 3 times more day 5 GQB with Kinetix Fertile quality sperm vs Infertile quality which also risks a 65% failure rate to produce day 5 GQB. A convenient way to implement this clinically is with use of cryopreserved specimens (Fig. 4). Because ejaculate quality varies within the same man, use of the Kinetix assay enables selection of an ejaculate prior to ICSI that contains an enriched population of FcR-positive sperm. In this way, injection from a sperm population enriched for function is made available to the embryologists without any manipulation of the sperm to be injected. Sperm can be cryopreserved, thawed, and injected according to established clinical procedures with the workflow shown below for use of the Kinetix assay, potentially creating several doses of known quality.

A limitation of this study was that all work was performed in one clinic, so this was a single-site study. Further, multi-center studies are therefore warranted.

In conclusion, the novel Kinetix assay offers a means to better understand underlying causes of recurrent cycle failures and unexplained infertility by providing insight into sperm-related factors, while also optimizing ICSI outcomes through selection of the most functionally competent ejaculate. Such an approach would provide patients with more information and higher chances of success. Higher success rates should also reduce psychological stress and dropout as well as encouraging recruitment. By enabling sperm functional assessment through expression patterns a ligand that functions as an Fc receptor, clinicians and patients can enter a new era of personalized medicine for male factor infertility treatment.

## Supplementary information

Below is the link to the electronic supplementary material.

Supplementary file1 (DOCX 30 kb)

## Declarations

### Data availability

The authors confirm that the data supporting the findings of this study are available within the article [and/or] its supplementary materials.
