# Supplementary material for: Use of a sperm FcR assay in intracytoplasmic sperm injection improves day 5 good quality blastocyst yield
Source: J Assist Reprod Genet. 2026 Jul 25;43(9):2985–93. doi: 10.1007/s10815-026-03978-z (PMC13731564; doi:10.1007/s10815-026-03978-z)
Supplement: Supplementary file 1 [file 10815_2026_3978_MOESM1_ESM.docx]

**Supplementary Tables**

**Supplementary Table 1.** Comparison of standard semen analysis parameters and male age according to Kinetix Assay score (Fertile, Subfertile or Infertile) based on sperm function.

| Parameter | Kinetix Score  Fertile (n=52) | Kinetix Score  Subfertile (n=69) | Kinetix Score  Infertile (n=17) |
| --- | --- | --- | --- |
| Ejaculate volume (ml) | 2.2 ± 0.9 | 2.5 ± 1.3 | 2.6 ± 0.2 |
| Sperm Count (10^6) | 91.5 ± 66.9 | 81.0 ± 56.2 | 60.4 ± 43.2 |
| Male Age (years) | 38.5± 5.6 | 38.4 ± 4.8 | 38.9 ± 4.1 |
| Rapidly Progressive Motility (%) | 56.5± 21.2 | 58.3±18.8 | 62.9±21.5 |

Function was determined by the temporal protein FcR expression pattern measured by assay and algorithm, which reflect capacitation activity. Data are expressed as Mean±SD with the exception of rapidly progressive motility expressed as percentage of rapidly progressive motile sperm (+4 and +3, WHO 6^th^ edition: rapidly progressive; ≥25 μm/s: spermatozoa moving actively, either linearly or in a large circle, covering a distance from the starting point to the end point, of at least 25 μm, or ½ tail length, in one second). Number of cycles in each group are also shown. No statistical differences were noted between male side parameters across Kinetix Score groups (one-way ANOVA).

**Supplementary Table 2.** Comparison of female parameters according to Kinetix Assay score (Fertile, Subfertile or Infertile) based on sperm function.

| Parameter | Kinetix Score  Fertile (n=52) | Kinetix Score  Subfertile (n=69) | Kinetix Score  Infertile (n=17) |
| --- | --- | --- | --- |
| Female Age (years) | 37 ± 4.3 | 37 ± 3.7 | 38 ± 5.2 |
| Anti-Mullerian Hormone, ng/ml | 2.6 ± 2.4 | 2.2 ± 2.1 | 2.7 ± 2.8 |
| Day 3 Follicle Stimulating Hormone IU/ml | 7.7 ± 3.4 | 8.7 ± 5.4 | 9.4 ± 4.3 |

Function was determined by the temporal protein FcR expression pattern measured by assay and algorithm, which reflect capacitation activity. Data are expressed as Mean±SD and number of cycles in each group are shown. No statistical differences were noted between female side parameters across Kinetix Score groups (one-way ANOVA).

**Supplementary Table 3.** Comparison of average yields on Day 6 and Day 7 of total and good quality blastocysts per 10 eggs fertilized normally in the Kinetix Assay sperm function groups of Fertile, Subfertile and Infertile.

| Total and Good Blastocyst Yields per Day | Kinetix Assay Groups | # cycles | Mean Blastocyst Yield ± SD per 10 Eggs (2PN) | *p (*one-way ANOVA) |
| --- | --- | --- | --- | --- |
| Day 6 Total | Fertile | 52 | 1.3 ± 2.0 | 0.94 |
|  | Subfertile | 69 | 1.2 ± 1.5 |  |
|  | Infertile | 17 | 1.2 ± 1.8 |  |
| Day 6 GQB | Fertile | 52 | 1.3 ± 1.4 | 0.68 |
|  | Subfertile | 69 | 1.4 ± 1.9 |  |
|  | Infertile | 17 | 1.0 ± 1.4 |  |
| Day 7 Total | Fertile | 52 | 0.3 ± 0.8 | 0.18 |
|  | Subfertile | 69 | 0.1 ± 0.5 |  |
|  | Infertile | 17 | 0.4 ± 1.2 |  |
| Day 7 GQB | Fertile | 52 | 0.5 ± 0.9 | 0.46 |
|  | Subfertile | 69 | 0.3 ± 0.9 |  |
|  | Infertile | 17 | 0.5 ± 1.2 |  |
